# Expected profitability, independence, and risk assessment of small farmers in the wave of GM crop collectivization——evidence from Xinjiang and Guangdong

**DOI:** 10.1080/21645698.2024.2445795

**Published:** 2025-01-09

**Authors:** Yu Pang, Helin Zou, Chunchun Jia, Chao Gu

**Affiliations:** a Research Center for Chinese Politics, School of Government, Peking University, Beijing, China; b School of Statistics and Mathematics, Central University of Finance and Economics, Beijing, China; c School of Business Administration, Nanjing University of Finance and Economics, Nanjing, Jiangsu, China

**Keywords:** Collective farming, developing countries, GM technology, independence, small farmers

## Abstract

As a longstanding and indispensable part of developing countries, small farmers face challenges brought by the dissemination of GM technology. Despite governments’ efforts to promote collective cultivation of GM crops through top-down policies aimed at enhancing small farmers’ production efficiency and market competitiveness, actual participation rates among small farmers in many developing countries remain low. This reflects a gap and mismatch between policy design and the actual needs of small farmers. Based on a survey and empirical analysis of 964 small farmers in Guangdong and Xinjiang, China, this study finds that small farmers’ acceptance of GM technology is influenced not only by expected profitability but also by factors such as their independence and risk assessment of the technology. The findings reveal that, first, small farmers’ expected profitability from GM technology and their perception of independent market adaptability positively influence their willingness to participate in collective GM crop farming. Independent market adaptability acts as a partial mediator in this relationship and is moderated by small farmers’ risk assessments of GM technology. Variables such as gender, age, education level, and farming experience do not show significant effects. This study enriches the theoretical frameworks related to technology acceptance, innovation and diffusion, livelihood strategies, and collective transformation among small farmers in developing countries. It provides scientific evidence for policymakers to design more effective and aligned policies concerning GM crops.

## 1. Introduction

Small farmer agriculture, with its unique advantages, plays a critical role in ensuring food security and maintaining rural social stability, making it a cornerstone of agricultural systems in developing countries.^1^ Despite the evolving social status of small farmers over time, as long as the fundamental contradiction between a large rural population and limited arable land resources remains unresolved, Small farmers farming will continue to persist in significant numbers over the long term. This remains a basic reality in most developing countries today.^2,3^ Simultaneously, as globalization and technological revolutions progress, small farmers face unprecedented changes, manifesting in the updates to technology, production methods, and transformations in agricultural organizational structures.^4,5^ Among these, various governments have implemented reforms that encourage small-scale farmers to transition toward collective cultivation, primarily through top-down policy initiatives.^6–9^ Collective farming, as an organizational model of agricultural production, seeks to enhance small farmers’ production efficiency and competitiveness by pooling resources such as land, capital, and technology. This approach offers advantages by reducing production costs, increasing responsiveness to market demands, and leveraging economies of scale to obtain more policy support and market opportunities.^10^ Genetically modified (GM) technology represents a critical tool in the development of collective farming for Small farmers. GM crops, through the introduction of traits such as pest tolerance, herbicide tolerance, and enhanced resilience under adverse conditions like drought,^11^ effectively reduce yield losses under biotic and abiotic stress, thereby increasing the likelihood of achieving potential yields. These attributes align closely with the objectives of collective farming, which seeks to maximize agricultural productivity. Recognizing these benefits, the Chinese government has actively promoted the collective cultivation of GM pest-resistant cotton (Bt cotton) since 1997.^12,13^

However, the actual outcomes of these policies have been less than ideal. Taking China and India as examples, China has promoted GM insect-resistant cotton since 1997,^14^ and by 2022, insect-resistant cotton accounted for 95% of China’s total cotton cultivation area, substantially increasing cotton yields. Nonetheless, more than 60% of China’s agricultural production is still conducted by individual Small farmers, and the collective farming level remains low. Insect-resistant cotton is scattered among individual farmers and fragmented plots, failing to form large-scale intensive production.^15,16^ Similarly, in India, where commercial GM insect-resistant cotton cultivation was approved in 2002, the cultivated area had reached 12.6 million hectares by 2019, accounting for 95% of the country’s total cotton planting area.^17,18^ However, approximately 86% of Indian farms are smaller than 2 hectares, with highly fragmented land and low levels of collectivization. Most Small farmers continue to rely on traditional individual farming methods, leading to higher production costs and difficulties in realizing the advantages of economies of scale.^19^ These figures illustrate the sub-optimal outcomes of GM crop collectivization policies and reveal a significant gap and mismatch between policy priorities and the actual needs of small farmers. So, what factors determine small farmers’ decisions in GM crop collectivization? Why is there a discrepancy between government policy priorities and small farmers’ needs, and what should those priorities be?

Building on this, the study conducted surveys and empirical analysis in two representative regions of commercial GM crop cultivation in China, Guangdong and Xinjiang. It delved into the actual needs of small farmers regarding resource endowments, market adaptability, and the process of technology promotion. Specifically, it examined their expectations of GM technology’s economic benefits, their preferences between independent operations and collective farming, and their assessments of associated risks in relation to their willingness to participate in collective GM crop cultivation. The results reveal that small farmers’ willingness to engage in collective farming of GM crops is influenced not only by expected profitability and technological risks but also significantly constrained by their sense of independence.

This study offers several key contributions to theoretical innovation: First, it enhances the comprehensive understanding of small farmers’ mechanisms for accepting GM technology. By integrating the Technology Acceptance Model (TAM),^a^^a^TAM, developed by Davis^50^ and based on the Theory of Reasoned Action (TRA), is primarily used to predict the acceptance and use of information technology. It focuses on two core variables – perceived usefulness and perceived ease of use – to forecast users’ attitudes, intentions, and actual usage behavior. Since its inception, TAM has evolved with extensions like TAM2, TAM3, and UTAUT, incorporating variables such as social influence, cultural factors, emotional experience, trust, and privacy. the Sustainable Livelihood Framework (SLF),^b^^b^SLF, proposed by the UK Department for International Development (DFID) in the 1990s, analyzes how individuals or groups use various resources to sustain livelihoods and respond to external environmental changes. It categorizes resources into five types of capital: natural, human, physical, financial, and social. SLF highlights that livelihood outcomes encompass not only economic benefits but also dimensions like environmental sustainability and social equity. It is widely applied in development studies, poverty alleviation, agriculture, and ecological conservation to assess resource endowments, identify livelihood risks, and formulate integrated intervention strategies. and the Adoption and Diffusion of Innovations Theory (ADIT).^c^^c^ADIT, proposed by Everett M. Rogers in 1962, examines how new technologies or innovations spread and are adopted within social systems. It identifies five key characteristics influencing diffusion: relative advantage (superiority over existing practices), compatibility (alignment with existing practices and values), complexity (ease of learning and use), trial ability (opportunity for small-scale testing), and observe ability (visibility of outcomes). ADIT also emphasizes the role of social networks, opinion leaders, and communication channels in technology dissemination, making it widely applicable in fields like agricultural technology, information systems, and medical innovations. This theoretical integration overcomes the limitations of a single framework, constructing a comprehensive analytical model that combines behavioral decision-making, resource endowments, and social network dissemination. Unlike studies solely relying on TAM to examine small farmers’ adoption behavior, this research incorporates SLF to enrich discussions on how resource endowments (e.g., natural and human capital) influence adoption. Simultaneously, by leveraging ADIT, it explores the role of technology characteristics and diffusion networks from the perspective of innovation dissemination. Second, the study provides a new perspective on the decision-making mechanisms in small farmers’ collective transformation in developing countries by using GM technology as a case study. Traditional theories of Small farmers collectivization mainly focus on agricultural institutional reforms (e.g., contract farming, agricultural co-ops), often overlooking the agency and unique needs of small farmers. This study reveals, through empirical surveys, the trade-offs and choices small farmers make between maintaining independent market adaptability and participating in collective GM crop cultivation. This provides a theoretical basis for understanding the core demands of small farmers in the process of collectivization. Lastly, the research offers scientific evidence for GM crop policy formulation. Policymakers should consider factors such as technological efficacy, market adaptation, and safety concerns when designing policies to better align with the actual needs of small farmers, thereby improving policy effectiveness and feasibility.

The structure of this article is as follows: Section 2 provides a literature review focusing on the TAM, SLF, and ADIT theories, the interaction between GM technology and small farmers, and the concept of collective farming, laying the theoretical foundation for the subsequent hypotheses. Section 3 presents the research hypotheses derived from the literature review. Section 4 describes the research design, including data sources, variable definitions, and methodologies. Sections 5 and 6 discuss the empirical results and their implications. Finally, Section 7 concludes with key findings and policy recommendations.

## 2. Literature Review and Background

### 2.1. GM Technology from the Perspective of Small Farmers

#### 2.1.1. Theoretical Frameworks for Small Farmers’ Adoption of GM Technology

Research on small farmers’ acceptance of technology frequently employs theoretical models such as TAM,^20^ SLF^21^ and ADIT.^22,23^ These theories provide a multidimensional foundation for understanding small farmers’ technology adoption behaviors, offering a rich array of perspectives.

TAM highlights the critical roles of perceived usefulness and perceived ease of use in shaping technology adoption intentions. In the context of GM technology, these two core variables explain how small farmers assess the potential economic benefits of the technology and its practical ease of implementation. If small farmers perceive GM crops as offering significant advantages, such as increased profitability (e.g., reduced pest damage, lower pesticide costs, and enhanced crop resilience), and believe that the technology is simple to operate and manage, their willingness to adopt GM technology is likely to increase. However, small farmers’ decisions are not solely based on the technology itself but are also influenced by resource constraints and the broader social environment, necessitating an analysis that goes beyond TAM’ s singular perspective.

Incorporating SLF allows a deeper exploration of the mechanisms behind small farmers’ profitability expectations. The resource endowments of small farmers – such as natural, human, financial, and social capital – significantly determine their acceptance of GM technology. For instance, financially well-resourced farmers are better positioned to absorb the high seed costs and market fluctuations, leading to higher adoption intentions. Conversely, farmers with limited natural capital may be more sensitive to GM technology’s potential to improve crop stability and resilience, making them more focused on its benefits. Additionally, social capital (e.g., connections with governments, cooperatives, or enterprises) can enhance trust in the technology, reduce information asymmetry, and boost confidence in GM crops, thereby increasing profitability expectations and adoption willingness.

From the perspective of ADIT, the relative advantage and trial ability of GM crops play a pivotal role in shaping small farmers’ profitability expectations. By observing demonstration plots or successful cases in neighboring areas, small farmers can directly perceive the economic benefits of GM technology, which significantly enhances their willingness to adopt it. Moreover, ADIT’ s dimensions of compatibility and complexity help explain hesitation in decision-making. If GM technology requires substantial integration with existing agricultural practices or involves high learning costs, small farmers may doubt its profitability. ADIT also emphasizes the importance of social networks and communication channels in technology diffusion. For example, through promotion by opinion leaders or the demonstration effects within a community, small farmers can more quickly receive positive information about the technology, further reinforcing their profitability expectations.

In agricultural contexts, TAM’ s perceived usefulness and ease of use were initially designed to analyze information technology adoption and do not fully account for the unique constraints faced by small farmers in production practices. Small farmers’ decisions are significantly influenced by livelihood constraints, meaning they often lack the complete freedom of choice available to typical consumers.^24^ Thus, in agricultural settings, TAM’ s variables should be reinterpreted as small farmers’ overall perceptions of the costs and benefits associated with a technology, encapsulated in profitability expectations. This reinterpretation retains TAM’ s core logic while better aligning with the realities of agricultural production.^25^

By integrating TAM, SLF, and ADIT, a more comprehensive understanding of small farmers’ adoption behavior can be achieved. SLF reveals how different types of capital affect small farmers’ perceptions of risks, such as safety, environmental, and market risks. ADIT complements this by emphasizing the direct impact of technology characteristics on adoption willingness, particularly the critical role of relative advantage in shaping profitability expectations. TAM provides a detailed analysis of the micro-level mechanisms through which technology characteristics influence adoption intentions. These three frameworks, while emphasizing different aspects, collectively form an integrated analytical model for understanding small farmers’ adoption of GM technology. Within this unified framework, small farmers’ adoption behavior is not only driven by their perceptions of GM technology’s economic benefits but is also shaped by their resource endowments and the social diffusion environment.

#### 2.1.2. Marginalized Farmers and Subsistence Farmers

When examining GM technology from the perspective of small farmers, scholarly research has primarily focused on two dimensions: Marginalized Farmers and Subsistence Farmers.^26^ These perspectives provide critical insights into the decision-making logic of small farmers in the process of adopting GM technology.

From the perspective of Marginalized Farmers, GM technology may exacerbate the marginalization of small farmers. Kuyek^27^ argued that the production and distribution of GM crop seeds are predominantly controlled by large agribusiness corporations. This monopolistic market structure can make it increasingly difficult for small farmers to access seeds and afford related costs. High costs and technological barriers reinforce small farmers’ subordinate status, making it harder for them to move away from subsistence agriculture. Such inequalities in resource allocation and market access may deepen their marginalization within the global agricultural system, leaving them in an even weaker position.

From the perspective of Subsistence Farmers, the decision to adopt GM technology often hinges on its potential to support Subsistence and mitigate risks. Giordano, Petropoulos, and Rouphael^28^ noted that the defining characteristic of subsistence farming – self-sufficiency – remains central to Small farmer economies, even as social environments evolve. This characteristic is particularly prominent in many developing countries.^29^ If GM technology is perceived as enhancing crop stability and resilience, thereby reducing risks from natural disasters, pests, and diseases, and ensuring food security, subsistence farmers may be more inclined to adopt it. However, if they perceive GM technology as introducing new uncertainties – such as risks to biodiversity, environmental health, or human health – they are more likely to avoid adopting it.

While TAM, as well as the perspectives of marginalized and subsistence farmers, emphasize different aspects, they all reflect the complex trade-offs that small farmers consider when adopting new technologies. These trade-offs involve balancing livelihood security with improving production efficiency. TAM and SLF emphasize economic benefits and market orientation, capturing small farmers’ pursuit of economic sustainability and increased income in a modern agricultural context. In contrast, the marginalized farmer perspective highlights the inequities and vulnerabilities small farmers face in accessing resources, technology, and market information within the context of globalization and commercialization. Meanwhile, the subsistence farmer perspective focuses on livelihood security and risk mitigation, analyzing the potential of GM technology to ensure food security and respond to environmental challenges. This process reveals that for both marginalized and subsistence farmers, adopting GM technology is not merely a technological decision but a complex process of adapting to a rapidly changing environment, maintaining livelihood security, addressing various risks, and achieving sustainable development. In making such decisions, small farmers must evaluate the long-term benefits and potential risks of the technology while considering their production capacity, resource accessibility, and market conditions. Especially in the context of increasing environmental variability, this dynamic adjustment ability becomes a crucial strategy for small farmers to maintain relative independence. They must not only navigate uncertainties in markets and technology but also adjust their decisions quarterly or annually based on changes in external environments and internal resource conditions. This process fundamentally reflects the multifaceted considerations small farmers make to balance independence and adaptability.

### 2.2. Collective Farming Among Small Farmers in Developing Countries

Historically, the top-down implementation of collective farming has seen limited success in countries like China, India, and many African nations. Poor organizational management, political interference, financial mismanagement, and internal corruption have constrained the effectiveness of these cooperatives.^30–32^ In collective initiatives such as contract farming and outsourcing schemes, buyers typically select Small farmers who are most likely to meet contract requirements, which results in the exclusion of other Small farmers.^33,34^ This exclusion exacerbates disparities among Small farmers, leading to unequal resource allocation and policy support. Many Small farmers, unable to benefit from collective farming, continue to rely on traditional independent production methods, which further trap them in low production efficiency and limited market competitiveness.^33,^^34^ Additionally, Small farmers in some countries face challenges such as uncertain land ownership, asymmetric market information, and inadequate technical extension and training, all of which pose significant barriers to their participation in collective farming.^35,36^

Current research primarily focuses on specific institutional reforms to address these issues. For example, some scholars have suggested strengthening land reforms to ensure clear land ownership for Small farmers, which would increase their willingness to invest and enhance production incentives.^37,38^ Additionally, by improving market transparency and information flow, Small farmers can better understand market demand and price fluctuations, enabling them to make more informed production decisions.^36^ In terms of technical extension and training, existing research emphasizes the role of both governmental and non-governmental organizations in providing resources and support to help Small farmers adopt modern agricultural technologies and improve production efficiency.^39,40^ At the same time, several studies highlight the critical role of cooperatives in addressing challenges faced by Small farmers.^6,41^ In successful cooperative models, such as those in the Netherlands and Finland, effective internal management and transparent financial systems have ensured the sustainable development of cooperatives and protected the interests of their members.^6^ Finally, for Small farmers who struggle to integrate into collective farming, contract farming and outsourcing schemes remain potential solutions.^33^ By entering into contracts with large companies, Small farmers can secure stable markets and income, thus reducing market risks.^42^

However, these studies often overlook Small farmers’ natural profit-driven behavior and their inherent operational limitations. Small farmers typically prioritize short-term gains to sustain their productivity, making them inclined to consider individual interests when faced with collective farming. Additionally, their small-scale operations, limited capital, and technological capacity often prevent them from taking a leading role in collective initiatives, leaving them vulnerable to marginalization. Ignoring these fundamental issues could not only undermine the efficiency and effectiveness of collective farming but also jeopardize Small farmers’ status and interests within the collective, thereby increasing the instability and sustainability of collective farming operations.^43,44^

## 3. Research Hypotheses

### 3.1. Analysis of the Impact of Small Farmers’ Profitability Expectations of GM Technology on Their Willingness to Participate in Collective GM Crop Cultivation

Profitability expectations are a key factor influencing small farmers’ willingness to adopt technology in their production decisions. TAM,^20^ emphasizes that an individual’s perceived usefulness and ease of use of a technology directly affect their adoption intentions. However, traditional TAM primarily addresses information technologies and does not fully account for the unique challenges and constraints faced by small farmers in agricultural production. By integrating SLF,^21^ it becomes evident that small farmers’ livelihood capital significantly impacts their profitability expectations. For instance, financially well-resourced farmers can better afford the high costs of seeds and mitigate risks associated with market fluctuations. Farmers with strong social capital, through connections with governments, cooperatives, or enterprises, can access more technical support and market information, enhancing their trust in GM technology.

From the perspective of ADIT,^22,23^ the relative advantages of GM technology – such as pest tolerance, reduced pesticide use, and improved crop resilience – enhance small farmers’ expectations of economic benefits. Additionally, social networks and dissemination channels, such as the influence of opinion leaders or demonstration effects within communities, increase small farmers’ recognition of and confidence in the technology, further boosting their profitability expectations. This view is supported by Wheeler^45^ and Qaim,^46^ who argue that small farmers skeptical of GM technology are often more concerned about potential risks and uncertainties, such as increased seed costs and dependency on large agribusiness corporations, which may erode profits. These concerns could offset the potential benefits of higher yields, ultimately undermining their profitability expectations.

Another critical aspect to consider is small farmers’ profit-driven orientation and operational limitations, which can be encapsulated as Independent Market Adaptation Ability. This concept refers to small farmers’ ability to maintain profitability as independent operators in the face of market changes. Due to their small scale, limited financial and technical capacity, small farmers often struggle to take a leading role in collective farming initiatives and are prone to marginalization. Nevertheless, their natural profit orientation and focus on short-term gains to sustain productivity lead them to carefully balance individual and collective interests when participating in collective farming. Independent market adaptation ability emphasizes the capacity to remain flexible and responsive to market changes within a collective farming framework.

Small farmers with strong market adaptation ability are more likely to identify market opportunities associated with GM crops, such as new demand, improved supply chain conditions, and potential high-value export opportunities. This capability increases their likelihood of participating in collective GM crop cultivation. As Minten et al.^47^ noted, maintaining the ability to respond to market risks within a collective farming framework allows small farmers to make more economically rational decisions, thereby reducing concerns about potential market acceptance risks associated with GM technology.

Based on this analysis, the following hypotheses are proposed:

Hypothesis 1: Small farmers’ profitability expectations of GM technology positively influence their willingness to participate in collective GM crop cultivation.

Hypothesis 2: Small farmers’ independent market adaptation ability positively influences their willingness to participate in collective GM crop cultivation.

### 3.2. Analysis of the Mediating Role of Small Farmers’ Independent Market Adaptation Ability

As previously discussed, Independent Market Adaptation Ability emphasizes the freedom to choose operational strategies and crop varieties outside the collective farming model, ensuring that small farmers, as independent business entities, can remain profitable in the face of market fluctuations. This independence is not limited to planting decisions; more importantly, it entails the flexibility to adjust according to market changes and the capability to identify and seize market opportunities. Small farmers with high independent market adaptation ability are better positioned to assess the potential yield benefits of GM technology and, by accurately predicting market demand, may opt for non-GM crops to effectively mitigate risks, thereby securing a competitive edge in the market. A lack of such ability may cause small farmers, even if they have positive expectations about the yield potential of GM technology, to hesitate when confronted with uncertainties in market acceptance, limited marketing channels, or the various challenges associated with technology adoption. Conversely, those with strong independent market adaptation ability are more likely to capitalize on market information, swiftly adjust to market changes, and establish broad marketing networks to reduce the risk of sales, making them more inclined to adopt GM technology.^48^ From the perspectives of marginalized and subsistence farmers, Independent Market Adaptation Ability is a crucial mediating variable in the adoption of GM technology. Enhancing this ability can help small farmers overcome external challenges such as market acceptance uncertainty and limited marketing channels. When small farmers have strong profitability expectations for GM technology, independent market adaptation ability enables them to participate in collective GM crop cultivation effectively.

Based on this analysis, the following hypothesis is proposed:

Hypothesis 3: Independent market adaptation ability mediates the relationship between small farmers’ profitability expectations of GM technology and their willingness to participate in collective GM crop cultivation.

### 3.3. Analysis of the Moderating Role of Small Farmers’ Risk Assessment of GM Technology

Risk assessment is an indispensable factor in small farmers’ decision-making process regarding participation in collective GM crop cultivation. As discussed earlier, small farmers evaluate GM technology by considering multiple potential risks, including production safety, ecological impacts, market uncertainties, and policy changes. These perceived risks significantly influence their trust and acceptance of the technology, thereby shaping their decisions.

Specifically, when small farmers perceive high risks associated with GM technology – such as substantial uncertainties or potential threats – they may lower their willingness to participate in collective farming, even if they possess strong independent market adaptation ability. A heightened perception of risk often leads small farmers to adopt conservative strategies to avoid potential losses. Conversely, when risk assessments are low and the technology is perceived as controllable or minimally impactful, small farmers with strong independent market adaptation ability are more likely to actively engage in collective farming and leverage their strengths to achieve higher profits.

**Figure 1.**
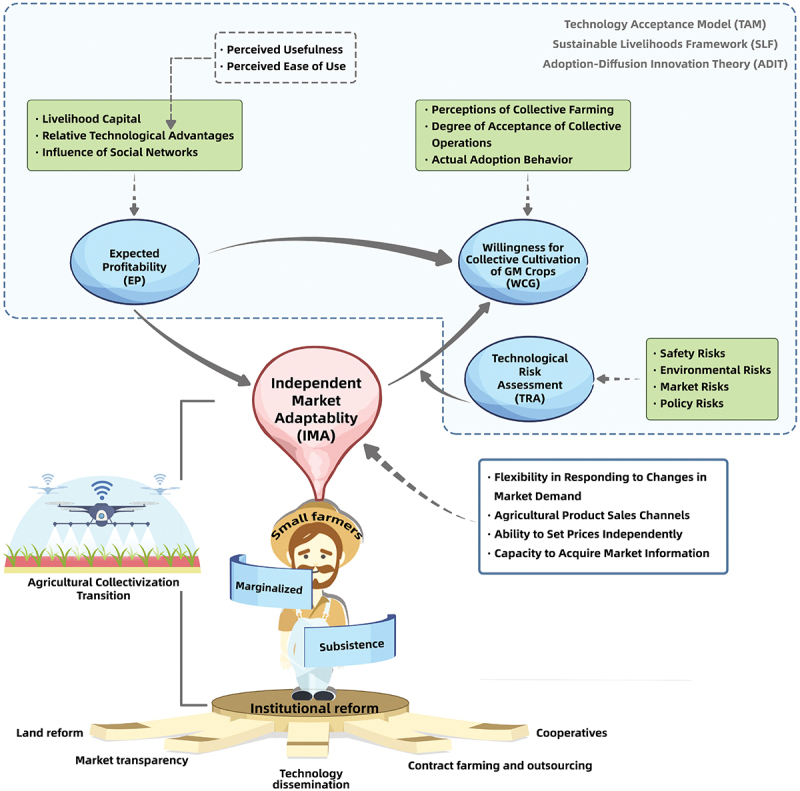
Theoretical analysis framework.

Thus, risk assessment serves as a negative moderator in the relationship between independent market adaptation ability and the willingness to participate in collective farming. High risk assessments weaken the positive impact of independent market adaptation ability on participation willingness, while low risk assessments strengthen this positive relationship. Theoretical analysis framework as shown in Figure 1.

Based on this analysis, the following hypothesis is proposed:

Hypothesis 4: Small farmers’ risk assessment of GM technology negatively moderates the relationship between independent market adaptation ability and their willingness to participate in collective GM crop cultivation.

## 4. Data Sources and Research Methods

### 4.1. Data Sources

China began the commercial cultivation of GM insect-resistant cotton in 1997 and GM papaya in 2006.^49^ The application of these two GM crops demonstrates the significant role of GM technology in Chinese agriculture. Xinjiang is one of the representative regions for the commercialization of GM cotton in China. It commenced commercial cultivation in 2000, and currently, the planting area accounts for nearly 70% of the national cotton cultivation area. Guangdong is one of the major papaya-producing regions in China, which has been researching and commercially cultivating GM papaya since 2006.^49^ By around 2010, it had achieved a 62% adoption rate and continued to grow. In both Xinjiang’ s GM cotton and Guangdong’s GM papaya cultivation, the collective farming model has provided a foundation for implementing GM technology.

Located in the northwest of China, Xinjiang adopts insect-resistant technology for GM cotton primarily to reduce pesticide use and improve cotton yield and quality. In this region, both large farms and small-scale farmers widely adopt GM cotton. Guangdong, located in the southeastern coastal area, primarily cultivates GM papaya to resist papaya ring-spot virus, with a significant number of small farmers participating in GM papaya cultivation. The selection of these two regions helps explore the application and promotion of GM technology in different crop sectors and small farmers’ attitudes and acceptance levels toward it. Additionally, GM cotton and GM papaya represent economic crops and food crops, respectively, which is significant for studying the application of GM technology in different types of crops.

Lastly, the markets faced by the two regions also differ. Xinjiang’s GM cotton industry closely serves the textile industry, which has strict requirements for cotton quality, yield, and cost. Xinjiang, located in northwest China, is near Central Asia and European markets, allowing its cotton products to enter international markets more conveniently through the Belt and Road Initiative. In contrast, Guangdong’s GM papaya is primarily aimed at the domestic consumer market, especially the fresh fruit market. Therefore, the selection of Xinjiang and Guangdong for the survey is based on their differences in geographical location, crop types, and market preferences, providing a comprehensive and diversified perspective for the research. This helps to deeply understand the desire of small farmers in different regions of China to maintain independent market adaptation capability when facing collective farming, as well as their attitudes and acceptance of GM technology.

The research team conducted field and online surveys in various areas of Guangdong and Xinjiang. Specifically, questionnaires were distributed to local small farmer operators through face-to-face interviews, telephone interviews, and online surveys. In face-to-face interviews, surveyors visited fields, markets, and rural residential areas to interact with respondents. Through telephone interviews, social media, and online survey platforms, surveyors contacted small farmer operators recommended by the initial respondents within their social networks. This diversified approach helps ensure the validity and reliability of the survey results.

The survey questionnaire consisted of 23 questions divided into five main sections, aiming to comprehensively assess small farmers’ (small-scale farmers, small family farms) yield expectations from GM technology, independent market adaptation capability, and concerns about the safety of GM technology. Detailed contents of the questionnaire are provided at the end of this paper.

**Figure 2.**
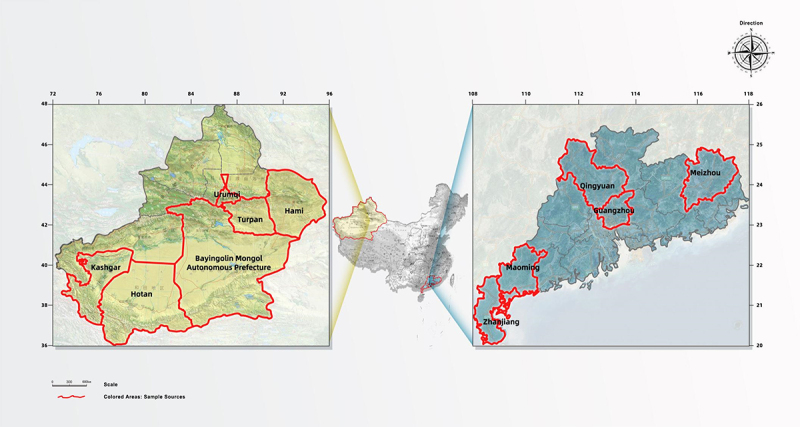
The location of the sample distribution.

The survey period spanned from January 2021 to August 2023, yielding a total of 1,064 questionnaires, with 964 valid responses, resulting in a 90.6% validity rate. Of the 964 responses, 10 were screened due to missing values for main variables, and the remaining 954 responses made up the final data. Among the participants, 616 were from Xinjiang and 348 from Guangdong. In 2021, 39 responses were obtained, with 21 from Xinjiang and 18 from Guangdong; in 2022, 99 responses were obtained, with 61 from Xinjiang and 38 from Guangdong; in 2023, 826 responses were obtained, with 534 from Xinjiang and 292 from Guangdong. The location of the visitors is as shown in Fig. 2.

### 4.2. Research Methods

#### 4.2.1. Variable Description

##### 4.2.1.1. Explained variable

Both Xinjiang and Guangdong have over a decade of development in the commercial cultivation of GM crops, with a foundational level of collective farming among local farmers. This provided a basis for defining the dependent variable, Willingness for Collective Cultivation of GM Crops (WCG). WCG was measured using a five-point Likert scale to assess the intensity of farmers’ willingness to engage in collective cultivation of GM crops. The scale, based on the works of Davis^50^ and Ali^51^ on technology acceptance and willingness to adopt GM crops, evaluates farmers’ perspectives on collective cultivation, acceptance of collective operations, and actual adoption behaviors. Respondents rated their agreement with certain statements, Respondents are asked to indicate their level of agreement with the following statements: *(1) ‘ I support the collective cultivation of GM crops.’; (2) ‘I believe collective cultivation of GM crops is beneficial to me.’; (3) ‘ I am willing to participate in the collective cultivation of GM crops.’*(see Appendix for details). Higher values indicate stronger willingness to participate in collective farming. The scale demonstrated high internal consistency reliability (Cronbach’s *α*  = 0.964), with WCG as a latent variable estimated from all four scale items.

##### 4.2.1.2. Explanatory variable

The independent variable, Expected Profitability (EP), measures Chinese small farmers’ overall understanding and evaluation of GM technology’s profitability. Using a five-point Likert scale, the study drew on scales from Davis,^50^ Guehlstorf,^52^ Areal,^53^ and Klümper,^54^ as well as authoritative discussions on GM crops’ impact on crop yields in developing countries by Qaim.^55^ The variable considers livelihood capital, relative technological advantages (perceived usefulness and ease of use), and social network effects. Respondents rated their agreement with certain statements across these four dimensions. Respondents rated their agreement with certain statements across these four dimensions: *(1) ‘I believe collective cultivation of GM crops can improve crop economic benefits.’; (2) ‘I believe neighbors or friends think that cultivating GM crops can increase profitability.’; (3) ‘I believe my current household resources (e.g., land, capital, labor) will be profitable if I adopted GM technology.’; (4) ‘ I believe not cultivating GM crops puts me at a disadvantage in terms of profitability compared to peers who cultivate them.’*(see Appendix for details). Higher values indicate greater confidence in GM technology’s profitability. This scale also showed high internal consistency reliability (Cronbach’s α = 0.859), with EP as a latent variable estimated from all four items.

##### 4.2.1.3. Mediating variable

The mediating variable, Independent Market Adaptability (IMA), was measured using a five-point Likert scale. Based on scales from Kondoh,^56^ Areal,^53^ and Chackal,^57^ and informed by Qaim’s^58^ research on small farmers’ economic resilience in dynamic markets, IMA evaluates flexibility in responding to market demand changes, product marketing channels, independent pricing ability, and access to market information. Respondents rated their agreement with certain statements across these four dimensions. Respondents are asked to indicate their level of agreement with the following statements: *(1) ‘I can identify and seize market opportunities brought by GM crops.’; (2) ‘I have consistently been able to independently select crop varieties suitable for market demand.’; (3) ‘ I can promptly decide the sales direction of my crops based on market needs.’; (4) ‘I can flexibly adjust my planting strategies according to market changes.’* (see Appendix for details). Higher values indicate greater market adaptability. The scale displayed strong internal consistency reliability (Cronbach’s *α* = 0.875), with IMA as a latent variable estimated from all four items.

##### 4.2.1.4. Moderating variable

The moderating variable, Technological Risk Assessment (TRA), was also measured using a five-point Likert scale. Based on scales from Guehlstorf,^52^ and Legge et al.^59^ on public risk assessments of GM technology, TRA assesses concerns over safety, environmental, market, and policy risks associated with GM crops. Respondents rated their agreement with certain statements reflecting their apprehensions about potential environmental and health impacts of GM crops. Respondents are asked to indicate their level of agreement with the following statements: *(1) ‘ I worry that GM crops may have negative effects on human health.’; (2) ‘I am concerned that safety issues with GM crops may affect their market acceptance.’; (3) ‘I am concerned that cultivating GM crops could have negative effects on the local ecological environment.’; (4) ‘I am worried that GM crop cultivation may impact the changes in government policies.’* (see Appendix for details). Higher values indicate greater risk perceptions. The scale demonstrated high internal consistency reliability (Cronbach’s α = 0.864), with TRA as a latent variable estimated from all four items.

##### 4.2.1.5. Demographic variables

Several demographic variables have been incorporated during data selection and variable choice. These include living area, gender, age, education level, household income level, family size, and whether GM crops have been previously cultivated. Specifically, the demographic variables are defined as follows (See the first part of the appendix). These variables may influence small farmers’ perceptions and behavioral responses to GM technology. Therefore, they have been considered and controlled in this study. The results of this study did not change in statistical significance before and after the inclusion of these statistical variables, so the final model did not retain these control variables.

#### 4.2.2. Analysis

In terms of statistical methods, this study primarily utilized IBM SPSS 26.0 and Mplus 8.3 software, with the latter employed to conduct confirmatory factor analysis.

First, following the recommendations of Kock^60^ and others, the Harman single-factor test indicated that four factors emerged without rotation, with the first factor explaining 33.759% of the variance – below the critical threshold of 40%. This suggested that common method bias was not a serious concern in the questionnaire scales.^61^

The main analysis of this study proceeded in four steps. First, we conducted descriptive statistical analyses of the demographic characteristics. Second, we tested the direct effect of EP on WCG. Third, we constructed a latent variable mediation model using latent EP as the independent variable, latent IMA as the mediator, and latent WCG as the dependent variable to explore the mediation effect. Finally, we applied a latent moderated mediation model to analyze the latent interaction between TRA and IMA on WCG, as well as the changes in the mediation effect of IMA. To test the moderating effect of TRA, a two-step procedure was required.^62^ First, we built a baseline null model (Model 0) that only assessed the moderating effect of TRA on WCG. The model fit was evaluated using the chi-square to degrees of freedom ratio (χ^2^/df), the Comparative Fit Index (CFI), the Tucker-Lewis Index (TLI), the Root Mean Square Error of Approximation (RMSEA), and the Standardized Root Mean Square Residual (SRMR).^63^ According to the recommendations for structural equation modeling by Wen et al.^64^ we adopted the following criteria as acceptable standards: CFI > 0.90, TLI > 0.90, RMSEA < 0.08, and SRMR < 0.08. Next, based on Model 0, we constructed Model 1 by adding the latent interaction term between TRA and IMA. We conducted a log-likelihood ratio test to evaluate whether Model 1 fit the data better than Model 0. If the log-likelihood ratio test showed significance and the latent interaction term significantly be associate with WCG, Model 1 was considered superior to Model 0. Statistical significance was set at a two-tailed p-value of 0.05.

## 5. Results

### 5.1. Descriptive Statistics and Correlations

The majority of participants were male, with ages predominantly ranging from 30 to 50 years. Notably, 77.5% of respondents had prior experience cultivating GM crops. The demographic characteristics of the respondents are summarized in Table 1.

**Table 1.** Demographic characteristics of respondents.

Variable	*N* (%)
Region	
Guangdong	346 (36.3%)
Xinjiang	608 (63.7%)
Gender	
Male	813 (84.5%)
Female	151 (15.5%)
Education Level	
Primary school or below	40 (4.2%)
Junior high school	149 (15.6%)
High school	246 (25.8%)
Associate degree	183 (19.2%)
Bachelor’s degree	223 (23.4%)
Master’s degree	84 (8.8%)
Doctoral degree	29 (3.0%)
Age	
Below 30 years	282 (29.6%)
30 to 50 years	447 (46.9%)
Above 50 years	225 (23.4%)
Family Size	
3 or fewer people	467 (48.9%)
More than 3 people	487 (51.1%)
Household Monthly Income	
Below 3500 RMB	199 (20.9%)
3500 to 7000 RMB	387 (40.6%)
7000 to 10,000 RMB	176 (18.4%)
10000 to 30,000 RMB	185 (19.4%)
Above 30,000 RMB	7 (0.7%)
Previous Cultivation of GM Crops	
Yes	739 (77.5%)
No	215 (22.5%)

A correlation analysis was conducted between the primary variables and demographic characteristics (see Table 2). The results indicated a significant positive correlation between EP and WCG (*r* = 0.213, *p* < .001).

**Table 2.** Pearson correlations between main variables. The four main variables (TRA, EP, IMA, TRA) were estimated as manifest variables, respectively using the mean value of all items in each scale. *p < .05, **p < .01, ***p < .001

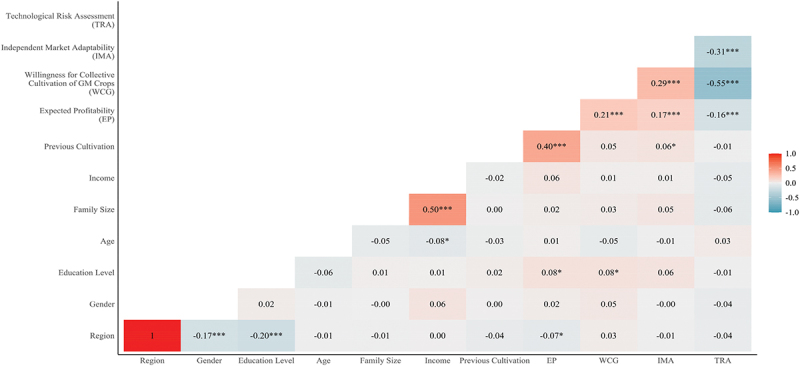

### 5.2. Direct Impact of Yield Expectations on GM Technology Adoption

The model tested the direct effect of EP on WCG, with EP as the independent variable and WCG as the dependent variable. The model exhibited a high level of fit with the data (*χ^2^/df*  = 3.617, CFI = 0.989, TLI = 0.982, RMSEA = 0.052, 90% CI = [0.037, 0.069], SRMR = 0.021). Analysis revealed a significant positive correlation between EP and WCG (*β* = 0.239, 95% CI = [0.170, 0.310], *p*  < 0.001), confirming H1.

### 5.3. Mediation Effect of Independent Market Adaptation Ability

**Figure 3.**
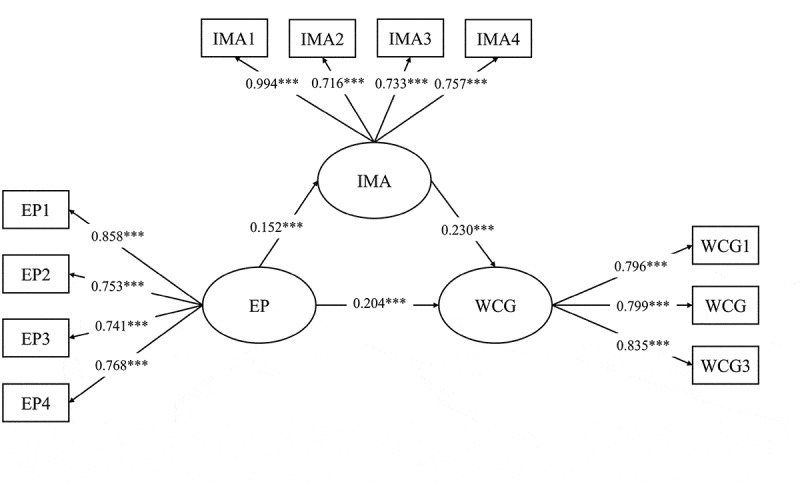
The latent mediation model.

The mediating role of IMA was assessed using a latent mediation model, as illustrated in Fig. 3. The model demonstrated good fit (χ^2^/df = 3.060, CFI = 0.984, TLI = 0.979, RMSEA = 0.046, 90% CI = [0.037, 0.056], SRMR = 0.035). The analysis identified a significant positive correlation between EP and IMA (β = 0.152, 95% CI = [0.090, 0.216], *p* < 0.001). Furthermore, IMA was found to positively influence WCG (β = 0.230, 95% CI = [0.153, 0.284], *p* < .001). When IMA was introduced as a mediator, the positive correlation between EP and WCG remained significant (β = 0.204, 95% CI = [0.132, 0.278], *p*  < 0.001). These indirect effects indicated that IMA significantly mediates the relationship between EP and WCG (β = 0.035, 95% CI = [0.018, 0.057], *p*  < 0.001), providing strong support for H2 and H3.

*p < .05, **p < .01, ***p < .001.

### 5.4. Moderating Effect of TRA

**Figure 4.**
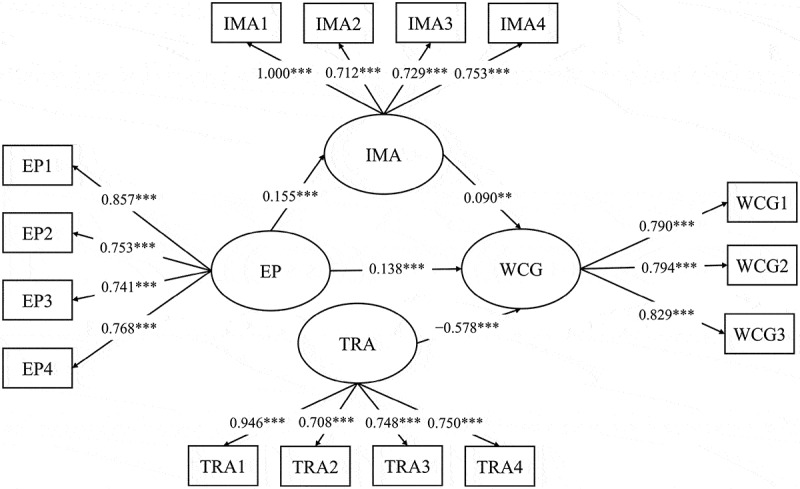
The null model without estimation of latent interactions.

**Figure 5.**
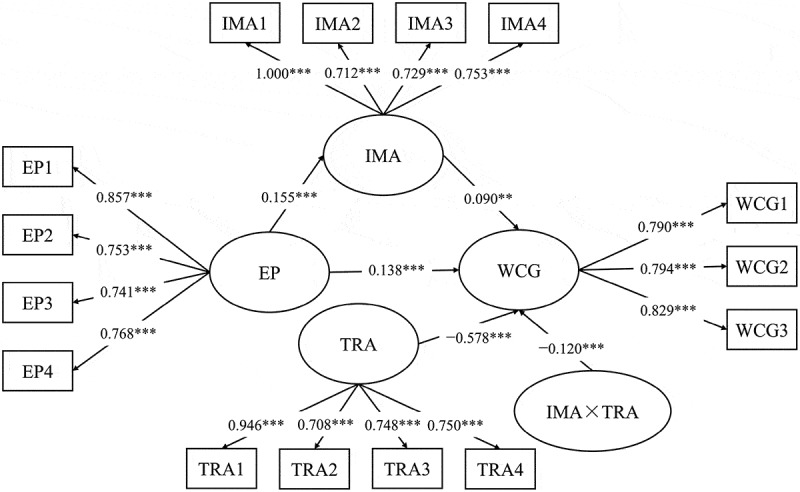
The latent moderated mediation model.

The moderating effect of TRA was analyzed using a latent moderated mediation model. Initially, Model 0, which excluded the latent interaction term, demonstrated good fit to the data (χ^2^/df = 3.283, CFI = 0.975, TLI = 0.969, RMSEA = 0.049, 90% CI = [0.043, 0.055], SRMR = 0.088) (see Fig. 4). Subsequently, Model 1 incorporated the latent interaction term between TRA and IMA (Independent Market Adaptation Ability) (see Fig. 5), resulting in a significant increase in log-likelihood (*D* = 13.020, *df*  = 1, *p* < 0.001). This indicated that Model 1 provides a better explanation of the data compared to Model 0. The results shown in Fig. 5 reveal that TRA moderates the mediating effect of IMA. Specifically, there is a significant interaction effect between TRA and IMA on WCG (β = −0.120, 95% CI = [−0.185, −0.055], p < 0.001).

*p < .05, **p < .01, ***p < .001.

*p < .05, **p < .01, ***p < .001.

When TRA was at a low level (i.e., one standard deviation below the mean), Fig. 5 indicates that higher levels of IMA (Independent Market Adaptation Ability) were positively associated with an increase in WCG (β = 0.162, 95% CI = [0.092, 0.233], *p* < 0.001). However, when TRA was at a high level (i.e., one standard deviation above the mean), the relationship between IMA and WCG becomed nonsignificant (β = −0.023, 95% CI = [−0.089, 0.044], *p* >0 .05).

**Figure 6.**
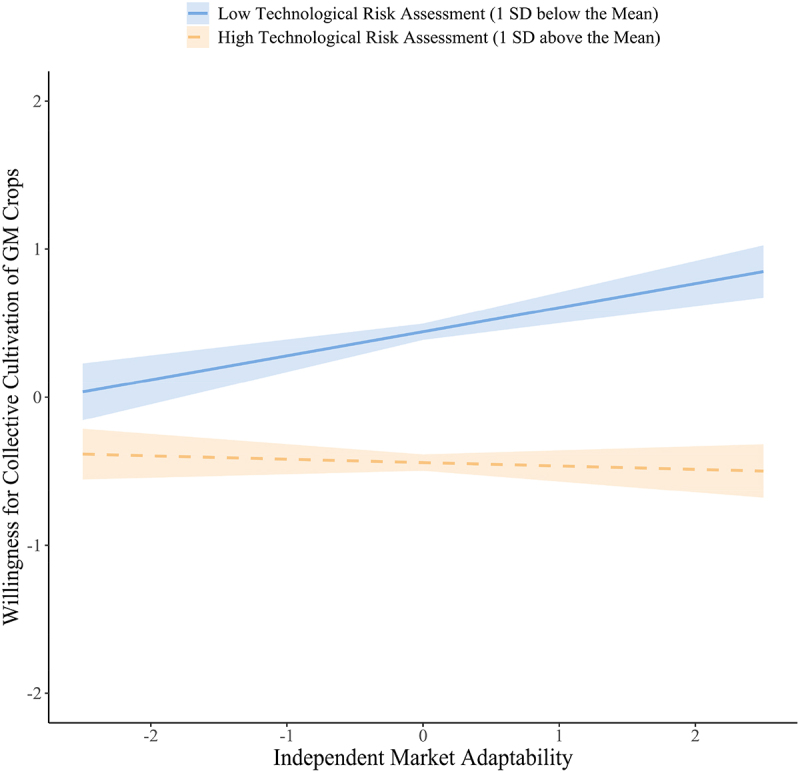
The simple slope for the moderating effects of concerns about the safety of GM technology.

Furthermore, the differences in the mediating effect of IMA (Independent Market Adaptation Ability) were analyzed at varying levels of TRA (Technological Risk Assessment). The results in Fig. 6 indicate that when TRA was at a low level (i.e., one standard deviation below the mean), the indirect effect of IMA was more significant (β = 0.029, 95% CI = [0.011, 0.047], *p* < 0.01). However, when TRA was at a high level (i.e., one standard deviation above the mean), the indirect effect was no longer significant (β = −0.004, 95% CI = [−0.016, 0.008], *p* >0 .05). These findings suggested that as TRA levels increase, the mediating effect of IMA on the relationship between EP (Expected Profitability) and WCG (Willingness for Collective Cultivation of GM Crops) gradually weakened, thereby supporting H4.

The present study also investigated the moderating role of TRA in both the mediating effects and the direct effects. A latent moderated mediation model was constructed, incorporating two interactions: the interactions between TRA and EP, and the interactions between TRA and IMA, both in relation to WCG. However, the results indicated that the new added interactions between TRA and EP were non-significantly associated with WCG (β = 0.013, 95% CI = [−0.056, 0.082], *p* >0 .05). Thus, TRA cannot moderate the direct effects.

## 6. Discussion

The findings indicate that small farmers’ EP of GM technology significantly influences their willingness to participate in collective cultivation (β = 0.204, *p* <0 .001), supporting H1. When small farmers perceive sufficient profit potential, they are more willing to engage in collective cultivation of GM crops. This, to some extent, drives the transformation and upgrading of Small farmer economies, enhances their endogenous development motivation, and facilitates their integration with professional cooperatives and agricultural service organizations. These findings align with the core perspectives of models such as TAM and ADIT, which emphasize that the perception of relative technological advantages is a critical driver of adoption behavior. Similarly, previous research has suggested that the adoption of GM crops effectively improves crop Subsistence rates, reduces reliance on inputs such as pesticides, and encourages farmers to participate in collective production systems.^65^ In resource-limited and competitive market environments, the high profitability expectations offered by GM technology serve as a key motivator for small farmers to adopt collective cultivation. These profitability expectations are not only a testament to the appeal of the technology itself but also reflect the farmers’ urgent desire to improve their standard of living. Hence, promoting GM technology should emphasize its potential profitability through targeted education and outreach to enhance farmers’ understanding and trust. The study also confirms that in the context of SLF, willingness to adopt GM technology is influenced not only by its economic benefits but also by variations in farmers’ resource endowments. For low-income Small farmers with limited livelihood assets, merely enhancing profitability expectations may not effectively drive adoption behavior.

Small farmers’ EP significantly influences their willingness to participate in collective cultivation of GM crops through the mediating role of IMA (β = 0.230, *p* <0 .001). This finding supports H2 and H3, while contrasting with certain existing studies. For instance, Peschard^66^ suggested that GM technology may increase Small farmers’ dependency on large-scale collective cultivation systems, thereby undermining their autonomy. However, this study reveals that the mediating effect of IMA allows Small farmers to maintain independent production models while treating collective cultivation more as a strategic choice. The primary goal of this collective approach is to help small farmers access more market information and technical support, thereby enhancing their independent market adaptability and competitiveness. Thus, government policies should encourage small farmers to find a balance between collective and independent operations, ensuring their autonomy and decision-making rights in the adoption of GM technology. This policy orientation would not only promote the sustainable participation of small farmers in collective GM crop cultivation but also provide an effective pathway for modernizing Small farmer agriculture in developing countries. Farmers with high market adaptability can better respond to technological uncertainties and market fluctuations, allowing them to capture demand shifts and adjust production strategies flexibly. Enhancing market adaptability alleviates farmers’ concerns about external market volatility and strengthens their active role in adopting GM technology through collective cultivation. Especially given the potential long-term benefits of GM crops, market adaptability becomes a crucial driver for Small farmer participation in collective cultivation.

Further analysis shows that TRA significantly moderates the relationship between IMA and willingness to participate in collective cultivation (β = −0.120, *p* <0 .001), supporting H4. Specifically, when small farmers perceive high potential risks from GM technology – such as ecological, safety, or policy uncertainties – even those with strong market adaptability may reduce their willingness to participate due to concerns over uncertainties. This suggests that farmers’ trust in the technology is a key factor influencing their decision-making, with risk perception partially undermining the positive effect of market adaptability. This finding aligns with ADIT, which posits that the “perceived risk” attribute can inhibit the diffusion of technology adoption. Interestingly, risk assessment does not moderate the direct effect of profitability expectations on participation in collective cultivation. According to IDT, technological attributes such as relative advantage (profitability expectations) and perceived risk represent distinct dimensions of adoption decisions. When farmers highly recognize the profitability potential of GM technology, they may still choose to participate despite certain perceived risks, prioritizing expected returns. Thus, risk assessment does not significantly moderate the relationship between profitability expectations and willingness to participate.

Regarding individual characteristics, this study found that control variables – such as location, gender, age, education level, household income, family size, and prior experience with GM crops – did not significantly influence the main model results, except for planting experience, which showed a notable impact on TRA during correlation analysis. This does not imply that these variables lack relevance in actual decision-making but rather underscores the dominant importance of EP, IMA, and TRA in influencing farmers’ participation decisions. These findings highlight the relative universality of the conclusions and suggest that promoting Small farmer participation in collective GM crop cultivation should focus more on technological and market independence factors while effectively addressing their concerns about GM crop risks.

Of course, this study is not without limitations. The data primarily come from surveys of small farmers in Guangdong and Xinjiang, China, and this method cannot fully eliminate the possibility of self-report bias. Future research can expand in several ways: First, by broadening the scope and increasing the diversity of samples. The current research focuses on two regions – Guangdong and Xinjiang. Future studies should include small farmers from more regions to ensure the general and representative of the findings. Second, conducting longitudinal studies. Short-term cross-sectional data may not fully capture the dynamic changes in Small farmers behavior under different policy environments and market conditions. Finally, future research can integrate advanced technologies such as big data analytic, artificial intelligence, and machine learning to develop more accurate models and predictive tools for analyzing and forecasting Small farmers decision-making under various policy and market conditions, providing scientific support for decision-making and policy development.

## 7. Conclusion

This study systematically investigated the impact of small farmers’ expected profitability of GM technology, independent market adaptability, and risk perceptions of GM crops on their willingness to participate in collective cultivation in developing countries. It extends the application of the TAM, SLF, and ADIT to the fields of Small farmer collective transformation and GM crop cultivation. The key conclusions are as follows:

First, small farmers’ expected profitability of GM technology significantly and positively influences their willingness to participate in collective cultivation of GM crops. GM technology enhances small farmers’ expectations of economic returns by reducing pesticide use, increasing yields, and improving stress tolerance. This indicates that high profitability expectations are a crucial motivator for small farmers to engage in collective cultivation.

Second, independent market adaptability plays a significant mediating role between profitability expectations and the willingness to participate in collective cultivation. Small farmers with stronger independent market adaptability can respond more flexibly to market changes, identify and seize market opportunities, and reduce reliance on collective cultivation. This suggests that even though collective cultivation of GM crops offers significant economic and technological advantages,^54^ small farmers still emphasize maintaining their market adaptability to ensure operational flexibility and economic independence within the collective framework.

Finally, small farmers’ risk perceptions of GM crops have a significant negative moderating effect on the influence of profitability expectations and independent market adaptability on their willingness to participate in collective cultivation. When small farmers perceive higher potential risks associated with GM crops – such as health, environmental, or policy uncertainties – their willingness to participate significantly decreases, even if their profitability expectations and independent market adaptability are strong. This finding indicates that risk perceptions to some extent weaken the appeal of GM technology, highlighting the importance of technological trust.

Based on the above research conclusions, this paper offers the following recommendations for policymakers and agricultural development practitioners, as shown in Table 3:

**Table 3.** Policy recommendations.

Policy Recommendations	Specific Measures	Expected Outcomes
Promote Participatory Policy Making	− Directly gather opinions from small farmers through meetings, questionnaires, etc. − Design policies that better align with the needs of small farmers	− Enhance the relevance and acceptance of policies −Increase small farmers’ trust in policy making
Formulate Differentiated Policy Measures	− Develop region-specific support programs based on local characteristics − Stable Regions: Strengthen the construction of cooperatives and provide incentives for collectivization − Volatile Regions: Support small farmers in enhancing independent market adaptability, such as flexible financing and market information services	− Adapt to market demands in different regions − Enhance competitiveness of small farmers in diverse markets
Strengthen Agricultural Extension and Education Services	− Expand the coverage of technology promotion and training programs − Improve technological understanding through demonstration fields and technical consultations − Provide scientific evidence and successful cases to high-risk perception groups to reduce information asymmetry	− Increase small farmers’ understanding and trust in GM technology − Assist small farmers in making rational decisions
Promote Mixed Farming Models	− Encourage small farmers to selectively participate in resource sharing and joint market sales − Provide support platforms that combine the economies of scale from collectivization with the advantages of independent Small farmer operations	− Enhance small farmers’ flexibility and economic independence − Achieve a balance between economies of scale and individual interests
Improve Mechanisms Addressing Environmental and Social Concerns	− Establish independent third-party certification mechanisms − Publicly disclose safety assessments of GM crops − Conduct risk monitoring and information disclosure, encourage community participation, and gradually eliminate public doubts about GM technology	− Enhance public trust in technology promotion − Reduce social controversies during the technology adoption process

## Supplementary Material

Supplemental Material

## Data Availability

The datasets generated and/or analyzed during the current study are not publicly available due related to other ongoing research but are available from the corresponding author on reasonable request.

## Appendix

**Table:**

Basic Information Section:	Gender: ________ (0 = Female, 1 = Male) Age: ________ (1 = Below 30 years, 2 = 30 to 50 years, 3 = Above 50 years) Education Level: ________ (1 = Primary school or below, 2 = Junior high school, 3 = high school, 4 = Associate degree, 5 = Bachelor’s degree, 6 = Master’s degree, 7 = Doctoral degree) Living Area: ________ (0 = Guangdong; 1 = Xinjiang) Household Income Level: ____________ (1 = Below 3500 RMB, 2 = 3500 to 7000 RMB, 3 = 7000 to 10,000 RMB, 4 = 10000 to 30,000 RMB, 5 = Above 30,000 RMB) Family Size: ________ (0 = 3 people or fewer, 1 = More than 3 people) Previous Cultivation of GM Crops: ________ (0 = No, 1 = Yes)
Please evaluate the following statements:	Q1: I believe collective cultivation of GM crops can improve crop economic benefits: 1 = Strongly Disagree, 2 = Somewhat Disagree, 3 = Neutral, 4 = Somewhat Agree, 5 = Strongly Agree Q2: I believe neighbors or friends think that cultivating GM crops can increase profitability. 1 = Strongly Disagree, 2 = Somewhat Disagree, 3 = Neutral, 4 = Somewhat Agree, 5 = Strongly Agree Q3: I believe my current household resources (e.g., land, capital, labor) will be profitable if I adopted GM technology:1 = Strongly Disagree, 2 = Somewhat Disagree, 3 = Neutral, 4 = Somewhat Agree, 5 = Strongly Agree Q4: I believe not cultivating GM crops puts me at a disadvantage in terms of profitability compared to peers who cultivate them: 1 = Strongly Disagree, 2 = Somewhat Disagree, 3 = Neutral, 4 = Somewhat Agree, 5 = Strongly Agree
	Q5: I can identify and seize market opportunities brought by GM crops: 1 = Strongly Disagree, 2 = Somewhat Disagree, 3 = Neutral, 4 = Somewhat Agree, 5 = Strongly Agree Q6: I have consistently been able to independently select crop varieties suitable for market demand: 1 = Strongly Disagree, 2 = Somewhat Disagree, 3 = Neutral, 4 = Somewhat Agree, 5 = Strongly Agree Q7: I can promptly decide the sales direction of my crops based on market needs: 1 = Strongly Disagree, 2 = Somewhat Disagree, 3 = Neutral, 4 = Somewhat Agree, 5 = Strongly Agree Q8: I can flexibly adjust my planting strategy next season based on market changes: 1 = Strongly Disagree, 2 = Somewhat Disagree, 3 = Neutral, 4 = Somewhat Agree, 5 = Strongly Agree
	Q9: I worry that GM crops may have negative effects on human health: 1 = Strongly Disagree, 2 = Somewhat Disagree, 3 = Neutral, 4 = Somewhat Agree, 5 = Strongly Agree Q10: I am concerned that safety issues with GM crops may affect their market acceptance: 1 = Strongly Disagree, 2 = Somewhat Disagree, 3 = Neutral, 4 = Somewhat Agree, 5 = Strongly Agree Q11: I am concerned that cultivating GM crops could have negative effects on the local ecological environment: 1 = Strongly Disagree, 2 = Somewhat Disagree, 3 = Neutral, 4 = Somewhat Agree, 5 = Strongly Agree Q12:I am worried that GM crop cultivation may impact the changes in government policies: 1 = Strongly Disagree, 2 = Somewhat Disagree, 3 = Neutral, 4 = Somewhat Agree, 5 = Strongly Agree
	Q13: I support the collective cultivation of GM crops: 1 = Strongly Disagree, 2 = Somewhat Disagree, 3 = Neutral, 4 = Somewhat Agree, 5 = Strongly Agree Q14: I believe collective cultivation of GM crops is beneficial to me: 1 = Strongly Disagree, 2 = Somewhat Disagree, 3 = Neutral, 4 = Somewhat Agree, 5 = Strongly Agree Q15: I am willing to participate in the collective cultivation of GM crops: 1 = Strongly Disagree, 2 = Somewhat Disagree, 3 = Neutral, 4 = Somewhat Agree, 5 = Strongly Agree
Please ensure to answer the questionnaire truthfully. Thank you for your participation and support!	
