# Supplementary material for: Expected profitability, independence, and risk assessment of small farmers in the wave of GM crop collectivization——evidence from Xinjiang and Guangdong
Source: GM Crops Food. 2025 Jan 9;16(1):97–117. doi: 10.1080/21645698.2024.2445795 (PMC11730364; doi:10.1080/21645698.2024.2445795)
Supplement: Supplemental Material [file KGMC_A_2445795_SM2753.docx]

**Appendix**

| Basic Information Section: | Gender: ________ (0 = Female, 1 = Male)  Age: ________ (1 = Below 30 years, 2 = 30 to 50 years, 3 = Above 50 years)  Education Level: ________  (1 = Primary school or below, 2 = Junior high school, 3 = High school, 4 = Associate degree, 5 = Bachelor's degree, 6 = Master's degree, 7 = Doctoral degree)  Living Area: ________ (0 = Guangdong; 1 = Xinjiang)  Household Income Level: ____________  (1 = Below 3500 RMB, 2 = 3500 to 7000 RMB, 3 = 7000 to 10000 RMB, 4 = 10000 to 30000 RMB, 5 = Above 30000 RMB)  Family Size: ________ (0 = 3 people or fewer, 1 = More than 3 people)  Previous Cultivation of GM Crops: ________ (0 = No, 1 = Yes) |
| --- | --- |
| Please evaluate the following statements: | Q1: I believe collective cultivation of GM crops can improve crop economic benefits.:  1 = Strongly Disagree, 2 = Somewhat Disagree, 3 = Neutral, 4 = Somewhat Agree, 5 = Strongly Agree  Q2: I would consider cultivating GM crops if recommended by neighbors or friends:  1 = Strongly Disagree, 2 = Somewhat Disagree, 3 = Neutral, 4 = Somewhat Agree, 5 = Strongly Agree  Q3: I believe my current household resources (e.g., land, capital, labor) support adopting new agricultural technologies:  1 = Strongly Disagree, 2 = Somewhat Disagree, 3 = Neutral, 4 = Somewhat Agree, 5 = Strongly Agree  Q4: I believe not cultivating GM crops puts me at a disadvantage in terms of yield compared to peers who cultivate them:  1 = Strongly Disagree, 2 = Somewhat Disagree, 3 = Neutral, 4 = Somewhat Agree, 5 = Strongly Agree |
|  | Q5: I can identify and seize market opportunities brought by GM crops:  1 = Strongly Disagree, 2 = Somewhat Disagree, 3 = Neutral, 4 = Somewhat Agree, 5 = Strongly Agree  Q6: I have consistently been able to independently select crop varieties suitable for market demand:  1 = Strongly Disagree, 2 = Somewhat Disagree, 3 = Neutral, 4 = Somewhat Agree, 5 = Strongly Agree  Q7: I can promptly decide the sales direction of my crops based on market needs:  1 = Strongly Disagree, 2 = Somewhat Disagree, 3 = Neutral, 4 = Somewhat Agree, 5 = Strongly Agree  Q8: I can flexibly adjust my planting strategy next season based on market changes:  1 = Strongly Disagree, 2 = Somewhat Disagree, 3 = Neutral, 4 = Somewhat Agree, 5 = Strongly Agree |
|  | Q9: I worry that GM crops may have negative effects on human health:  1 = Strongly Disagree, 2 = Somewhat Disagree, 3 = Neutral, 4 = Somewhat Agree, 5 = Strongly Agree  Q10: I am concerned that safety issues with GM crops may affect their market acceptance:  1 = Strongly Disagree, 2 = Somewhat Disagree, 3 = Neutral, 4 = Somewhat Agree, 5 = Strongly Agree  Q11: I am concerned that cultivating GM crops could have negative effects on the local ecological environment:  1 = Strongly Disagree, 2 = Somewhat Disagree, 3 = Neutral, 4 = Somewhat Agree, 5 = Strongly Agree  Q12: I am worried that changes in government policies may impact the profitability of GM crop cultivation:  1 = Strongly Disagree, 2 = Somewhat Disagree, 3 = Neutral, 4 = Somewhat Agree, 5 = Strongly Agree |
|  | Q13: I support the collective cultivation of GM crops:  1 = Strongly Disagree, 2 = Somewhat Disagree, 3 = Neutral, 4 = Somewhat Agree, 5 = Strongly Agree  Q14: I believe collective cultivation of GM crops can make my agricultural products easier to sell:  1 = Strongly Disagree, 2 = Somewhat Disagree, 3 = Neutral, 4 = Somewhat Agree, 5 = Strongly Agree  Q15: I am willing to participate in the collective cultivation of GM crops:  1 = Strongly Disagree, 2 = Somewhat Disagree, 3 = Neutral, 4 = Somewhat Agree, 5 = Strongly Agree |
| Please ensure to answer the questionnaire truthfully. Thank you for your participation and support! | |
